# Virtual care use among older immigrant adults in Ontario, Canada during the COVID-19 pandemic: A repeated cross-sectional analysis

**DOI:** 10.1371/journal.pdig.0000092

**Published:** 2023-08-02

**Authors:** Janette Brual, Cherry Chu, Jiming Fang, Cathleen Fleury, Vess Stamenova, Onil Bhattacharyya, Mina Tadrous

**Affiliations:** 1 Women’s College Hospital Institute for Health System Solutions and Virtual Care, Toronto, Ontario, Canada; 2 ICES, Toronto, Ontario, Canada; 3 Institute of Health Policy, Management and Evaluation, Dalla Lana School of Public Health, University of Toronto, Toronto, Ontario, Canada; 4 Department of Family and Community Medicine, University of Toronto, Toronto, Ontario, Canada; 5 Leslie Dan Faculty of Pharmacy, University of Toronto, Toronto, Ontario, Canada; University of Modena and Reggio Emilia, Policlinico di Modena, ITALY

## Abstract

The critical role of virtual care during the COVID-19 pandemic has raised concerns about the widening disparities to access by vulnerable populations including older immigrants. This paper aims to describe virtual care use in older immigrant populations residing in Ontario, Canada. In this population-based, repeated cross-sectional study, we used linked administrative data to describe virtual care and healthcare utilization among immigrants aged 65 years and older before and during the COVID-19 pandemic. Visits were identified weekly from January 2018 to March 2021 among various older adult immigrant populations. Among older immigrants, over 75% were high users of virtual care (had two or more virtual visits) during the pandemic. Rates of virtual care use was low (weekly average <2 visits per 1000) prior to the pandemic, but increased for both older adult immigrant and non-immigrant populations. At the start of the pandemic, virtual care use was lower among immigrants compared to non-immigrants (weekly average of 77 vs 86 visits per 1000). As the pandemic progressed, the rates between these groups became similar (80 vs 79 visits per 1000). Virtual care use was consistently lower among immigrants in the family class (75 visits per 1000) compared to the economic (82 visits per 1000) or refugee (89 visits per 1000) classes, and was lower among those who only spoke French (69 visits per 1000) or neither French nor English (73 visits per 1000) compared to those who were fluent in English (81 visits per 1000). This study found that use of virtual care was comparable between older immigrants and non-immigrants overall, though there may have been barriers to access for older immigrants early on in the pandemic. However, within older immigrant populations, immigration category and language ability were consistent differentiators in the rates of virtual care use throughout the pandemic.

## Author summary

When the COVID-19 pandemic began, healthcare systems pivoted from in-person to virtual care to maintain physical distancing. Studies have shown that virtual care use became much more frequent during the pandemic as a result. What we do not know is whether virtual care is being used equitably, that is, whether everybody has fair access to the resource. This can be a big issue particularly amongst older adults, who are often battling several diseases and use healthcare frequently. Many older adults are immigrants who may face challenges in accessing healthcare due to reasons such as limited language fluency and resource support. Our study found that older adult immigrants aged 65 and above living in Ontario, Canada had lower use of virtual care initially, but their use eventually caught up with non-immigrants as the pandemic progressed. We also found that older adult immigrants from the family class had lower virtual care use compared to those from the economic, refugee, or other immigration classes. Additionally, immigrants who were not fluent in English had lower use compared to those who were fluent. These results show that virtual care access remains an issue for vulnerable minorities and steps should be taken to ensure these groups are receiving adequate care.

## Introduction

In March 2020, the World Health Organization declared the outbreak of the novel coronavirus disease (COVID-19) a pandemic [1], and as a result, health systems around the world had to adapt quickly in order to continue providing access to healthcare services. To accommodate safety protocols of physical distancing and to reduce virus transmission, health care shifted from predominantly in-person care to remote care using virtual modalities such as telephone and video-based visits, asynchronous messaging, and digital applications. In response, the Ontario government issued temporary billing codes which expanded on the types of visits that physicians are eligible to bill for, specifically telephone and video visits [2]. Prior to the pandemic, physician reimbursement for virtual care was restrictive in that it only covered videoconferencing on specific government-run platforms [3] and was mainly targeted towards rural patients. A recent study examining the period before and during the COVID-19 pandemic found that virtual care was used modestly prior to the pandemic (less than 5% of all ambulatory visits), but has been maintained between 50–80% of all ambulatory visits in Ontario, Canada during the pandemic [4].

As virtual care becomes more common and innovations in digital health continue to be developed post-pandemic, there is growing concern that the digital divide will widen and disadvantage the most vulnerable populations [5,6], particularly older immigrants. Older adults have complex health needs, higher rates of chronic disease, disabilities, and comorbidities, and are among the highest users of the healthcare system [7,8]. Immigrants in Canada have been especially affected by the pandemic, with immigrants being more likely to work jobs with higher exposure to COVID-19 and have greater economic vulnerability. Recent immigrants are also more likely to have worse mental health levels compared to established immigrants or non-immigrants [9]. Older immigrants in Canada are even more vulnerable to breakdowns in the system, and may face additional challenges when accessing healthcare, including cultural differences, discrimination, language barriers, literacy, health beliefs, and spatial isolation [10–13]. In addition, some barriers to access for older adult populations, such as physical or mental disabilities, inexperience or discomfort with technology, or lack of digital equipment [14–16] may be even more pronounced among immigrants who migrated at an older age and have poor social determinants of health upon arriving in Canada. Furthermore, some recent immigrants may face further health decline with increasing years of residency in Canada and poor access to health care [17]. While early reports during the pandemic showed that the rates of virtual care visits were highest among older adults compared to younger groups [18], it remains unclear how immigration status affected virtual care use. To our knowledge, few published studies have investigated the use of virtual care or telemedicine services among immigrant populations, and the ones that have were mostly limited to data from individual clinics or practices. A systematic review found that non-immigrants were more likely to attend remote consultations in primary care [19] while another US study based in a family medicine clinic found that refugees were less likely to use telemedicine [20].

This paper aims to describe virtual care use in older immigrant populations in Ontario overall, as well as across various immigrant sub-groups.

## Methods

### Ethics statement

We received approval from the Women’s College Hospital Research Ethics Board for use of the IRCC–Permanent Residents Database. Ethics review through a research ethics board was not required for use of the other administrative databases for the purposes of this study as authorized under section 45 of Ontario’s Personal Health Information Protection Act [21].

### Data sources

We used linked and coded population-based health databases from ICES (formerly the Institute for Clinical Evaluative Sciences) to examine the utilization of virtual care among the older adult patient population. ICES is an independent, non-profit research institute whose legal status under Ontario’s health information privacy law allows it to collect and analyze health care and demographic data, without consent, for health system evaluation and improvement.

The databases used for our study included: (1) The Immigration, Refugee, and Citizenship Canada (IRCC) Permanent Residents Database, which has demographic data of all immigrants to Canada who become permanent residents including information on country of birth, citizenship and country of residence, admission category and landing date or age at landing; (2) Registered Persons Database (RPDB), which contains demographic information of all patients covered under the Ontario Health Insurance Plan; (3) Ontario Health Insurance Plan (OHIP), which includes information on all health services delivered by physicians to Ontario patients who are eligible for coverage; (4) various ICES-derived chronic disease registries. Databases were linked using unique encoded identifiers and analyzed at ICES.

### Study design and setting

This analysis reports on virtual care use among older immigrant populations and is part of a larger study of virtual care use among older adults before and during the COVID-19 pandemic. The results of the broader study are reported elsewhere [18]. We conducted a population-based, repeated cross-sectional study of virtual ambulatory visits among older immigrants (age 65 years and older) residing in Ontario, Canada with valid Ontario Health Insurance Plan (OHIP) healthcare coverage. Individuals who were non-Ontario residents, had an invalid health card number or were residing in long-term care were excluded from analysis. Visits were identified using OHIP administrative claims data before and during the COVID-19 pandemic, from January 1, 2018 to March 29, 2021. All OHIP billing codes used to identify and calculate the rates of virtual visits are available in the S1 Appendix.

### Patient characteristics

Various subgroups of patients were identified using the relevant databases, including patient demographics and immigration records. ‘Immigrant’ refers to those who immigrated to Canada during or after 1985. ‘Non-immigrant’ subgroups refer to any individual not recorded in the immigration database and includes both Canadian-born residents and immigrants who arrived in Canada before 1985. ‘Recent immigrant’ includes immigrants who arrived during or after 2016 and ‘Earlier immigrant’ includes immigrants who arrived between 1985 and 2015. Among the immigration classes, the family category involves reunification of Canadian citizens or permanent residents with close family members such as spouses, common-law or conjugal partners, and dependent children [22]. Economic immigrants are selected based on the **National Occupational Classification** according to skills and ability to contribute to the Canadian economy. This category may include immigrants in the business category (entrepreneurs, investors and self-employed), as well as skilled and unskilled workers. The refugee category refers to immigrants who received status for refugee and asylum protection. Finally, the ‘other’ category refers to immigrants and permanent residents who do not belong to the other immigration categories, and may include immigration by humanitarian and compassionate reasons.

We used the Rurality Index of Ontario (RIO) [23] to determine rurality among the study population, comparing urban (RIO score < 40) versus rural (RIO score ≥ 40) residence. The Postal Code Conversion File (PCCF) was used to convert all patient postal codes to neighborhood income quintiles. A number of ambulatory care sensitive conditions (ACSC) were also reported and included chronic obstructive pulmonary disease (COPD), heart failure, asthma, hypertension, angina, and diabetes. Hospitalizations due to ACSC conditions are often used as an indicator of health system performance. ACSC can be managed through timely and effective disease management within primary care [24] and greater comorbidity of these conditions could increase the risk of hospitalization if not well managed.

We further categorized patients based on their use of virtual care (high vs low) to compare health status, immigrant status, and health services utilization. High users were defined as patients who received two or more virtual visits after March 14, 2020 until end of study period, while low users were defined as patients who received one or no virtual visits after March 14, 2020 until end of study period. For this analysis, March 14, 2020 was considered the start date of the COVID-19 pandemic as it was the day that new temporary billing codes were introduced by the Ontario government which expanded physician reimbursement of telemedicine services (including telephone calls) in response to the pandemic. The threshold of two visits was used after reviewing the distribution of frequency of virtual visits per patient, with approximately 44% of the cohort having 0–1 virtual visit during the study period and the remainder having 2 or more.

### Analysis

Rates of virtual visits were calculated for each week (from Sunday to Saturday) from January 1st, 2018 to March 29th, 2021. The denominator for each week’s rate calculation included all residents of Ontario who were age 65 and above during the week and eligible for healthcare services in Ontario (i.e., OHIP-insured). We summarized the overall rates of virtual visits within the older immigrant population, comparing rates to the non-immigrant population and across the following subgroups: recent versus non-recent immigrant status, immigration category (economic, family, refugee and ‘other’ category), and Canadian language ability (English and French). All analyses were performed in SAS 9.4 (SAS Institute) [25].

## Results

### Overall patient characteristics by immigration status

Baseline patient characteristics are summarized in **Table 1** comparing older adult immigrants and non-immigrants. Overall, a greater proportion of older adults were women compared to men (54.2% versus 45.8%). Immigrants were younger with 73.7% compared to 66.6% of non-immigrants in the youngest-old category (65–74 years). A higher proportion of older immigrants lived in urban areas compared to non-immigrants (99.0% versus 89.1%), and they had lower incomes, with 26.3% of older immigrants compared to 18.0% of non-immigrant older adults in the lowest income quintile. For both immigrant and non-immigrant patient populations, the top previously diagnosed disease conditions were hypertension, diabetes, and mental health. The number of patients with hypertension and mental health was similar between these two groups, however, more immigrants had diabetes compared to non-immigrants (42% versus 30%).

**Table 1:** Patient characteristics for older immigrant and non-immigrant adults in Ontario, Canada.

Characteristic	Total N = 2,282,798	Immigrant n = 271,075	Non-Immigrant n = 2,011,723	Standardized Difference^a^
Age, n(%)				
65–74	1,539,340 (67.4)	199,780 (73.7)	1,339,560 (66.6)	0.156
75–84	567,625 (24.9)	57,168 (21.1)	510,457 (25.4)	0.102
85+	175,833 (7.7)	14,127 (5.2)	161,706 (8.0)	0.114
Sex, n(%)				
Female	1,233,462 (54.0)	146,974 (54.2)	1,086,488 (54.0)	0.004
Male	1,049,336 (46.0)	124,101 (45.8)	925,235 (46.0)	0.004
Neighbourhood income quintile, n(%)				
1	432,945 (19.0)	71,331 (26.3)	361,614 (18.0)	0.202
2	469,631 (20.6)	61,869 (22.8)	407,762 (20.3)	0.062
3	458,824 (20.1)	56,250 (20.8)	402,574 (20.0)	0.018
4	439,791 (19.3)	46,581 (17.2)	393,210 (19.5)	0.061
5	476,247 (20.9)	34,560 (12.7)	441,687 (22.0)	0.245
Rurality, n(%)				
Urban	2,060,282 (90.3)	268,240 (99.0)	1,792,042 (89.1)	0.426
Rural	200,182 (8.8)	2,203 (0.8)	197,979 (9.8)	0.41
Region, n(%)				
Central	690,994 (30.3)	154,034 (56.8)	536,960 (26.7)	0.642
East	599,098 (26.2)	56,981 (21.0)	542,117 (26.9)	0.139
North	144,377 (6.3)	1,119 (0.4)	143,258 (7.1)	0.358
Toronto	174,752 (7.7)	26,179 (9.7)	148,573 (7.4)	0.081
West	673,577 (29.5)	32,762 (12.1)	640,815 (31.9)	0.492
Chronic conditions, n(%)				
Hypertension	1,583,965 (69.4)	194,053 (71.6)	1,389,912 (69.1)	0.055
Diabetes	721,699 (31.6)	114,210 (42.1)	607,489 (30.2)	0.25
Mental Health	751,608 (32.9)	79,545 (29.3)	672,063 (33.4)	0.088
Asthma	223,451 (9.8)	23,670 (8.7)	199,781(9.9)	0.041
Angina	166,430 (7.3)	20,158 (7.4)	146,272 (7.3)	0.006
CHF	202,396 (8.9)	17,881 (6.6)	184,515 (9.2)	0.096
COPD	188,974 (8.3)	11,536 (4.3)	177,438 (8.8)	0.185
Dementia	68,528 (3.0)	6,934 (2.6)	61,594 (3.1)	0.03

^a^ Standardized difference greater than 0.1 is considered statistically significant.

### Immigrant characteristics by high vs low virtual care user group

Among immigrants residing in Ontario, most arrived under the family immigration category (50.4%), followed by immigrants under the economic category (32.7%) with the fewest in the refugee and ‘other’ immigration categories (13.7% and 3.1%, respectively) (**Table 2**). In terms of Canadian language ability (English and French), more than half of immigrants reported being able to speak English (53.3%), followed by immigrants who spoke neither English nor French (44.3%). Few immigrants were able to speak both English and French (1.6%) or French only (0.8%). Most immigrants arrived before January 1^st^, 2016 (97.8%) with few recent immigrants (2.2%). 78% of the immigrant population were high users of virtual care during the COVID-19 pandemic. The mean number of ACSC was 1.50±0.98 and 1.09±0.91 in high and low users of virtual care, respectively. The average number of outpatient visits was higher among high users (3.81±4.37) compared to low users (1.42±3.18). Similarly, more high virtual care users had at least one ED visit (13.5% vs 8.2%) and hospitalization (4.8% vs 2.8%) than low users.

**Table 2:** Patient characteristics and healthcare utilization among older immigrant adults only, by virtual care use in Ontario, Canada.

Virtual Care Group^a^	Total N = 271,075	High User N = 211,304	Low User N = 59,771	Standardized Difference^c^
Immigration Category, n (%)				
Family	136,755 (50.4)	108,926 (51.5)	27,829 (46.6)	0.1
Economic	88,608 (32.7)	67,672 (32.0)	20,936 (35.0)	0.064
Refugee	37,144 (13.7)	28,109 (13.3)	9,035 (15.1)	0.052
Other	8,568 (3.2)	6,597 (3.1)	1,971 (3.3)	0.01
Immigrant Language Ability, n (%)				
Both French and English	4,377 (1.6)	3,375 (1.6)	1,002 (1.7)	0.006
English	144,479 (53.3)	113,001 (53.5)	31,478 (52.7)	0.016
French	2,034 (0.8)	1,480 (0.7)	554 (0.9)	0.025
Neither French nor English	120,019 (44.3)	93,304 (44.2)	26,715 (44.7)	0.011
Not Stated	166 (0.1)	144 (0.1)	22 (0.0)	0.014
Time of Arrival in Canada^b^, n (%)				
Earlier immigrant	265,233 (97.8)	206,674 (97.8)	58,559 (98.0)	0.011
Recent immigrant	5,842 (2.2)	4,630 (2.2)	1,212 (2.0)	0.011
Number of ACSC, mean (SD)	1.41 (0.98)	1.50 (0.98)	1.09 (0.91)	0.425
Number of virtual care visits, mean (SD)	5.94 (6.30)	7.45 (6.37)	0.6 (0.49)	1.518
Proportion of virtual care visits out of all ambulatory visits, mean (SD)	61.3 (31.0)	69.1 (23.1)	33.7 (38.9)	1.109
Number of outpatient visits in 6 months prior, mean (SD)	3.28 (4.25)	3.81 (4.37)	1.42 (3.18)	0.625
Visited ED in 6 months prior, n(%)	33,456 (12.3%)	28,561 (13.5%)	4,895 (8.2%)	0.172
Hospitalized in 6 months prior, n(%)	11,831 (4.4%)	10,180 (4.8%)	1,651 (2.8%)	0.108

^a^ High virtual care use is defined as two or more virtual visits after March 14, 2020, and low users, defined as zero or one virtual visits after March 14, 2020.

^b^ “Recent immigrant” refers to immigrants who arrived in Canada after Jan 1, 2016.

^c^ Standardized difference greater than 0.1 is considered statistically significant.

### Virtual care use: Immigrants versus Non-Immigrants

**Fig 1:**
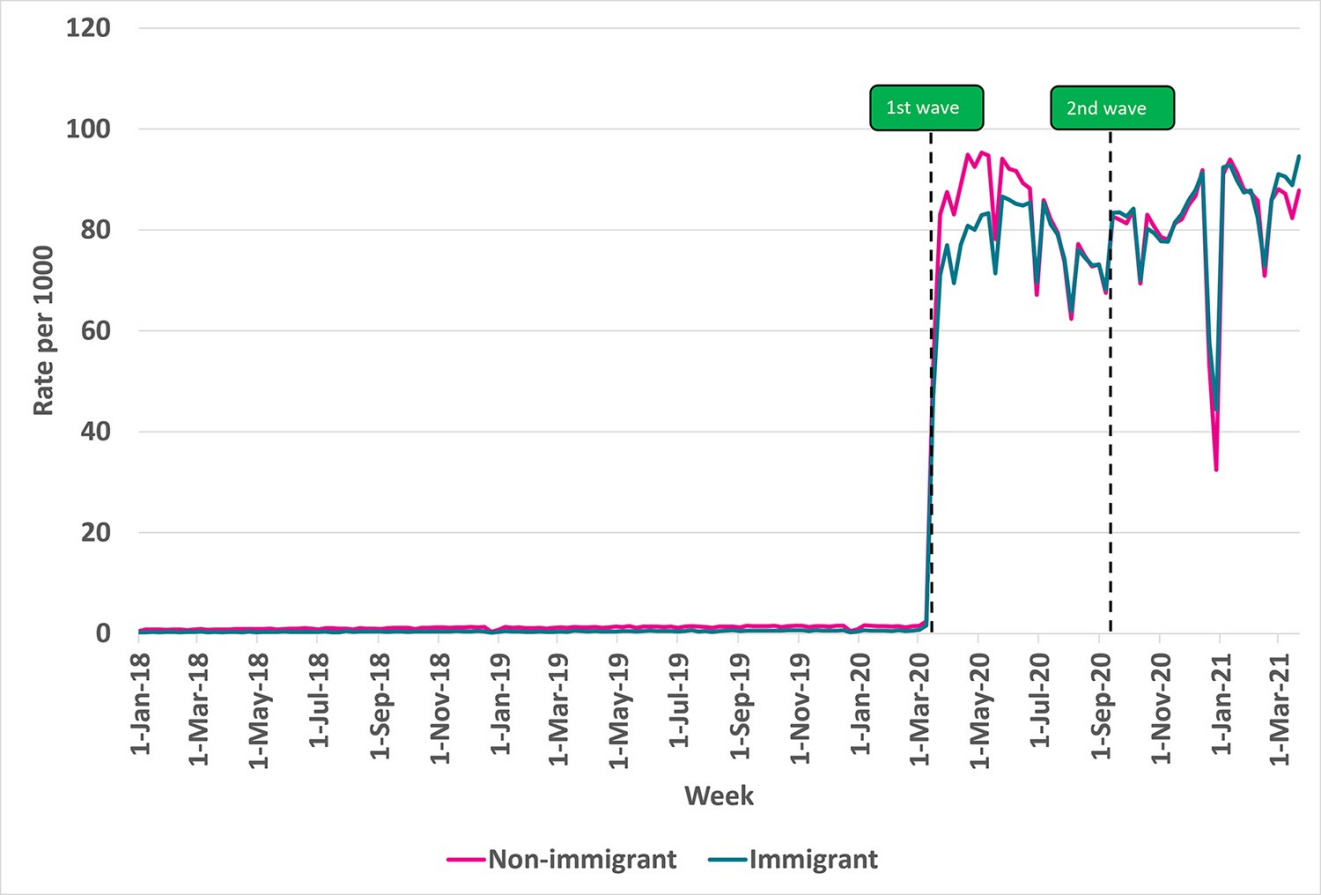
Rate of virtual visits per 1000 eligible older adult patients in Ontario: Immigrant versus non-immigrant subgroups, 2018–2021. Note: Non-immigrants may include immigrants who arrived in Canada before 1985.

**Fig 1** compares weekly rates of virtual care use between older immigrant and non-immigrant populations. Both reported a significant increase in the rates of virtual care visits at the start of the COVID-19 pandemic, but the rates in non-immigrants were greater than those for immigrants, with average weekly rates (per 1000) of 86 and 77, respectively. However, as the pandemic continued onward, virtual care use between both groups became similar, with average rates (per 1000) of 79 and 80, respectively.

### Virtual care use by Immigration status

**Fig 2:**
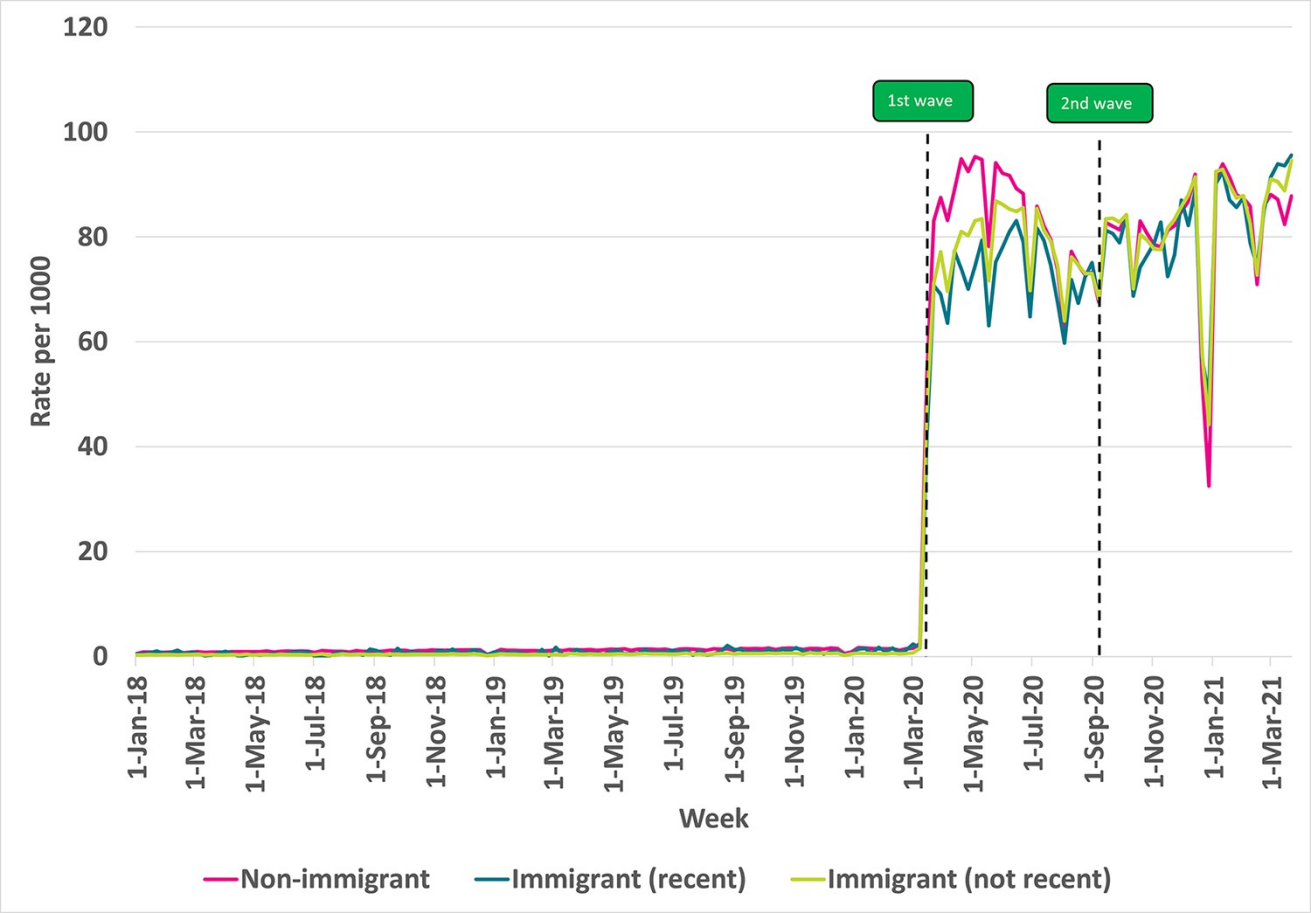
Rate of virtual visits per 1000 eligible older immigrant patients in Ontario by recency of immigration, 2018–2021. Note: Recent = immigrated after Jan 1, 2016; Non-immigrants may include immigrants who arrived in Canada before 1985.

As shown in **Fig 2**, during the first wave of the pandemic, earlier immigrants had slightly higher rates of virtual care use compared to recent immigrants (arrived after January 1, 2016), with average weekly rates (per 1000) of 78 and 71, respectively, although both groups had lower rates compared to non-immigrants (86 visits per 1000). By the summer of 2020 and onwards into the pandemic, rates for both earlier and recent immigrants increased to similar rates as non-immigrants, with average weekly rates (per 1000) of 80, 78, and 79, respectively.

### Virtual care use by Canadian Language Ability

**Fig 3:**
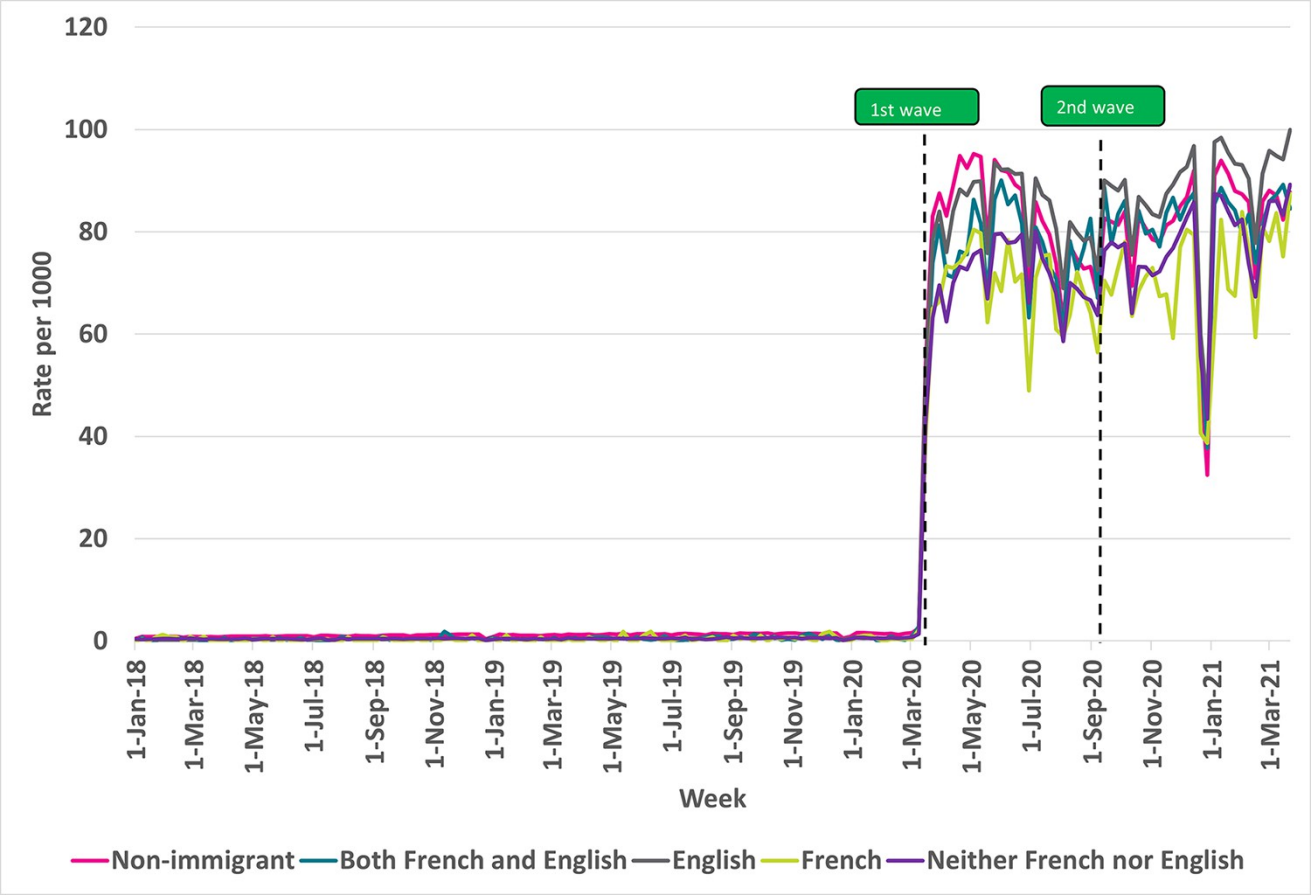
Rate of virtual visits per 1000 eligible older immigrant patients in Ontario by Canadian language ability, 2018–2021. Note: Non-immigrants may include immigrants who arrived in Canada before 1985.

**Fig 3** shows weekly rates of virtual care use according to Canadian language ability among non-immigrant and immigrant subgroups. All groups reported a significant increase in weekly rates of virtual care visits at the start of the COVID-19 pandemic. From the first wave onwards into the pandemic, virtual care use was highest among non-immigrants, immigrants with English language ability, and immigrants who speak both French and English, with average weekly rates (per 1000) of 81, 86, and 79, respectively. However, those who speak neither English nor French or only speak French had lower virtual care use, with average weekly rates (per 1000) of 69 and 73, respectively.

### Virtual care use by Immigration category

**Fig 4:**
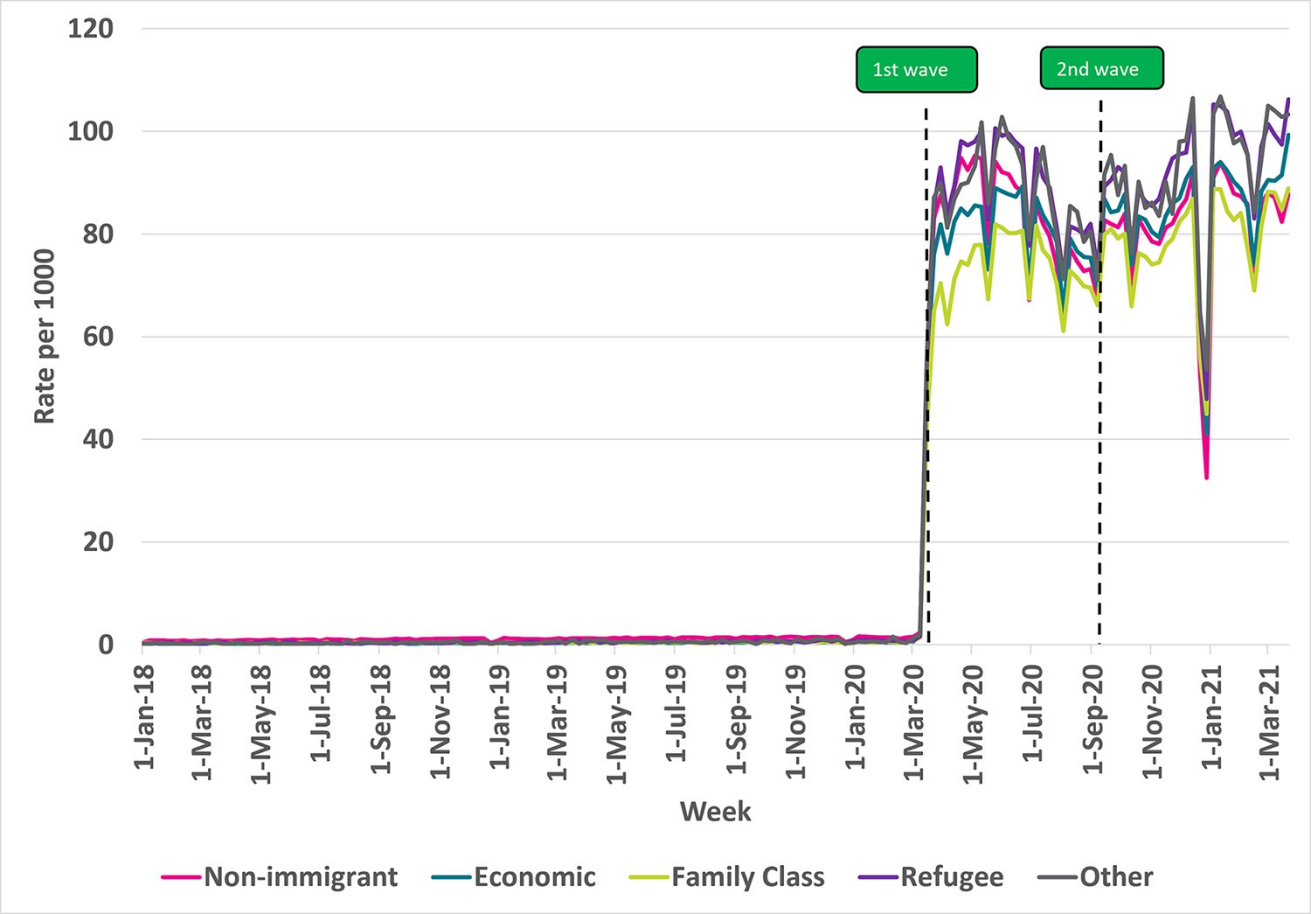
Rate of virtual visits per 1000 eligible older immigrant patients in Ontario by immigration category, 2018–2021. Note: Non-immigrants may include immigrants who arrived in Canada before 1985.

All immigration categories reported a significant increase in weekly rates of virtual care visits during the COVID-19 pandemic as shown in **Fig 4**. From the first wave onwards, virtual care use was highest among refugees and those in the ‘other’ immigration categories, with average weekly rates (per 1000) of 89 for both. These rates were followed by non-immigrants and immigrants in the economic immigration category (e.g., skilled and unskilled workers), with average weekly rates (per 1000) of 81 and 82, respectively. Older immigrants in the family category used virtual care the least, with average weekly rates (per 1000) of 75.

## Discussion

Overall, this study showed that virtual care use was lower among older immigrant populations when the pandemic began compared to non-immigrant populations—however, as the pandemic progressed the rates between these groups converged and became similar. Among older immigrant populations, immigration admission category and language ability were found to be consistent differentiators in the rates of virtual care use throughout the pandemic in Ontario. Despite the consistent finding of increased virtual care uptake across all demographic groups assessed, there remains potential equity issues with adoption within the older adult immigrant population.

While there was an overall reduction in health service use at the start of the pandemic as health systems shifted to virtual methods, there was a lag in virtual care adoption among older immigrant populations. Of particular concern are older immigrants who had more chronic or severe health issues, where having limited access to care at the start of the pandemic may have had detrimental effects on their health resulting in greater use of care as the pandemic progressed. Reasons for this apparent lagged difference in virtual care uptake for immigrants is unknown, however these findings suggest that although there are increasing healthcare needs among older immigrant populations, there may be significant barriers when accessing virtual care services. As previously noted, some of these barriers may be related to language, socioeconomic status, and other immigrant-related experiences. [10–13] Specifically, studies have found that older age and lower education levels are often associated with lower digital health literacy and lower virtual care adoption. [26,27] However, when looking at the proportion of virtual versus in-person visits out of all ambulatory visits in our study (see S4 Appendix figures), we found that non-immigrants had a higher proportion of in-person visits (and therefore lower proportion of virtual visits) compared to immigrants. These results may suggest that non-immigrants were more likely to be offered and/or to choose in-person rather than virtual care.

Within the immigrant cohort, virtual care use was lowest among those in the family immigration category when compared to other categories, while virtual care use was higher among the refugee and economic classes of older immigrants. Lower rates of virtual care use among older immigrants of the family class was a surprising finding, as individuals in this group, by definition, would likely have family members or strong social support to assist them with attending virtual visits. However, shifts in family dynamics over time (death of spouse, children moving with the labour market, transitional care from home-setting, etc.), particularly among earlier immigrants, can impact the availability of economic and social resources in later life, resulting in poorer access to care [28]. Another possible explanation is that many newer immigrant families may be of lower socioeconomic status [29], which could present as a barrier to accessing virtual care, particularly with limited access to internet or digital tools (i.e. digital device), in addition to non-technical barriers such as digital health literacy, comfort with digital technology, and lack of trust towards digital devices [30]. Interestingly, despite family class immigrants having the lowest rates of virtual care use, their proportion of virtual to in-person visits was highest compared to other groups (see S4 Appendix figures), perhaps suggesting that they sought less care during this time and when they did seek care, they were more likely to be offered and/or to choose virtual visits compared to in-person. Economic class immigrants reported the second lowest rates of virtual care use–although the reason for this finding is unclear, it may be related to these individuals being younger and healthier than other immigrants. Canadian immigration and settlement are not monolithic experiences, and the intersectionality of realities experienced by immigrants during the pandemic and onward can greatly impact their access to health care services overall, including virtual care.

Our analyses also found that the lowest rates of virtual care visits during the pandemic were among immigrants who only spoke French, and immigrants who had neither English nor French-language proficiencies. Immigrants who are unable to communicate in the dominant language can experience several issues relating to patient safety, appropriate treatment, and quality of care [31]. The provision of healthcare services in French (outside of Quebec) and non-official or minority languages is a significant resource challenge, and many immigrants experience difficulties understanding their healthcare providers, and may find the quality of translation services inconsistent or availability of services unreliable [32]. Ad hoc interpreters and use of digital translation tools such as Google translate, while helpful, may not be adequate for patients to receive the same quality of care. A few studies have cited the impact of language barriers in impeding the uptake of virtual care. [20,33,34] Many of the communication challenges faced by patients who are unable to speak the dominant language in healthcare during in-person encounters likely translate to or worsen with virtual care, regardless of modality, i.e. telephone or video. However, if used optimally, virtual care could alternatively provide a means to connect patients with a healthcare provider who are proficient in their language.

Another potential barrier to virtual care uptake in general is the heterogeneity of reimbursement policies for virtual care, and in some cases, the lack of reimbursement at all. In several jurisdictions outside of Canada, vast differences in the reimbursement of digital health modalities compared to in-person care has hindered the widespread implementation of digital health delivery. [35–38] In Canada, although virtual care has become universal due to the pandemic, reimbursement policies differ from province to province [39], and in some provinces there has yet to be reimbursement policies implemented for newer modalities of virtual care such as asynchronous messaging and remote monitoring.

Findings from this study show that virtual care provided comparable access to healthcare for many immigrant groups and should be continued as an option for care, but efforts should be made to leverage virtual modalities to decrease the equity gap in access rather than widen it.

### Limitations

The generalizability of this descriptive study is limited by some constraints with our choice of analysis and data. First, the lack of clinical information that accompanies the use of administrative data requires acknowledgment. Lack of patient-level medical history or previous healthcare use limits our ability to contextualize patients’ use of virtual care as it relates to their health care needs. Second, with the introduction of new COVID-19 virtual care billing codes in Ontario, reimbursement was permitted for both telephone and video visits but did not allow for distinguishing modality used during the visits. As such, we were unable to determine whether the virtual visits identified from the database were delivered via telephone or video. Third, our single factor analyses were unable to examine the intersectionality of the immigrant experience and its association with virtual care access. Therefore, while there may be significant barriers for individuals who are a recent immigrant, non-English speaker, and have low socioeconomic status, we were unable to show how these intersecting identities may impact their use of virtual care. Fourth, we linked to the IRCC Permanent Residents database to identify immigrant status, however this database only includes immigrant data from 1985 onward. Any persons who immigrated to Ontario prior to 1985 would be excluded from the database and as such, we acknowledge that the use of the term “non-immigrant” may also include people who arrived in Ontario before 1985 [40]. Similarly, our definition of “recent immigrant” as arriving after Jan 1, 2016 at the time of analysis, may not be an accurate representation of landing experiences in Canada, particularly for immigrants who migrated at an older age. However, the literature supports the notion that immigrants living in Canada for at least 15 to 20 years have similar health statuses and behaviours as Canadian-born residents. [41,42] Fifth, we were unable to account for inter-provincial migration of immigrants, as the IRCC Permanent Residents database excludes records of immigrants who first landed in a province other than Ontario. Inter-provincial migration can be influenced by different regional economic, political and social factors, as well as being a function of age, behavioural, and lifestyle preferences. [43] However, older immigrants–particularly family class immigrants–are least likely to migrate after arrival in Canada [44], and we can assume inter-provincial migration would be relatively low among this cohort, since selective internal migration often occurs among immigrants of working age. Lastly, the analyses here looked at the short-term shift in virtual care use among older immigrant populations as a result of the COVID-19 pandemic, however future evaluations should continue to monitor virtual care use in older immigrants post-pandemic.

## Conclusion

To our knowledge, this is the first study that examines virtual care use among older immigrant populations, particularly during the COVID-19 pandemic. Our results suggest that there is a clear link between immigration status and virtual care use. In response to the pandemic, public health measures facilitated the rapid uptake of virtual care among the total older adult population, and while there was a lagged difference in use between immigrant and non-immigrant populations in the early months of the pandemic, these rates converged as time progressed. These results suggest that virtual care was able to address gaps in access for older adults broadly, but that the adjustment period from in-person to virtual care, and the learning curve among older immigrant groups, may be longer. Future research should focus on the early access barriers to virtual care that are experienced by vulnerable subgroups such as recent immigrants, as this has important policy implications in a country with many immigrants such as Canada. Additionally, the impact of language barriers and opportunities on the uptake of virtual care should be further researched.

## Supporting information

S1 Appendix Descriptions of virtual care billing codes. (DOCX)

S2 Appendix Flowchart of virtual visits included in study. (DOCX)

S3 Appendix Flowchart of ambulatory visits included in study. (DOCX)

S4 Appendix Proportion of virtual vs in-person visits out of all ambulatory visits, by subgroup. (DOCX)
