# Supplementary material for: Virtual care use among older immigrant adults in Ontario, Canada during the COVID-19 pandemic: A repeated cross-sectional analysis
Source: PLOS Digit Health. 2023 Aug 2;2(8):e0000092. doi: 10.1371/journal.pdig.0000092 (PMC10395820; doi:10.1371/journal.pdig.0000092)
Supplement: S1 Appendix — (DOCX) [file pdig.0000092.s001.docx]

**S1 Appendix. Descriptions of virtual care billing codes**

| **Billing code** | **Description** |
| --- | --- |
| B100 | Hosted video visit: First telemedicine patient encounter premium |
| B200 | Hosted video visit: Subsequent telemedicine patient encounter premium |
| B099 | Direct-to-patient video visit: Tracking code |
| B103 | Hosted video visit: Patient attending at a patient host site |
| B203 | Direct-to-patient video visit |
| K080 | Minor assessment of a patient by telephone or video, or advice or information by telephone or video to a patient's representative regarding health maintenance, diagnosis, treatment and/or prognosis |
| K081 | a. intermediate assessment of a patient by telephone or video, or advice or information by telephone or video to a patient's representative regarding health maintenance, diagnosis, treatment and/or prognosis, if the service lasts a minimum of 10 minutes; or b. psychotherapy, psychiatric or primary mental health care, counselling or interview conducted by telephone or video, if the service lasts a minimum of 10 minutes |
| K082 | Psychotherapy, psychiatric or primary mental health care, counselling or interview conducted by telephone or video per unit (unit means half hour or major part thereof) per unit |
| K083 | Specialist consultation or visit by telephone or video payable in increments |
