# Supplementary figures and images for: Virtual care use among older immigrant adults in Ontario, Canada during the COVID-19 pandemic: A repeated cross-sectional analysis
Source: PLOS Digit Health. 2023 Aug 2;2(8):e0000092. doi: 10.1371/journal.pdig.0000092 (PMC10395820; doi:10.1371/journal.pdig.0000092)

**S2 Appendix. Flowchart of virtual visits included in study**

**
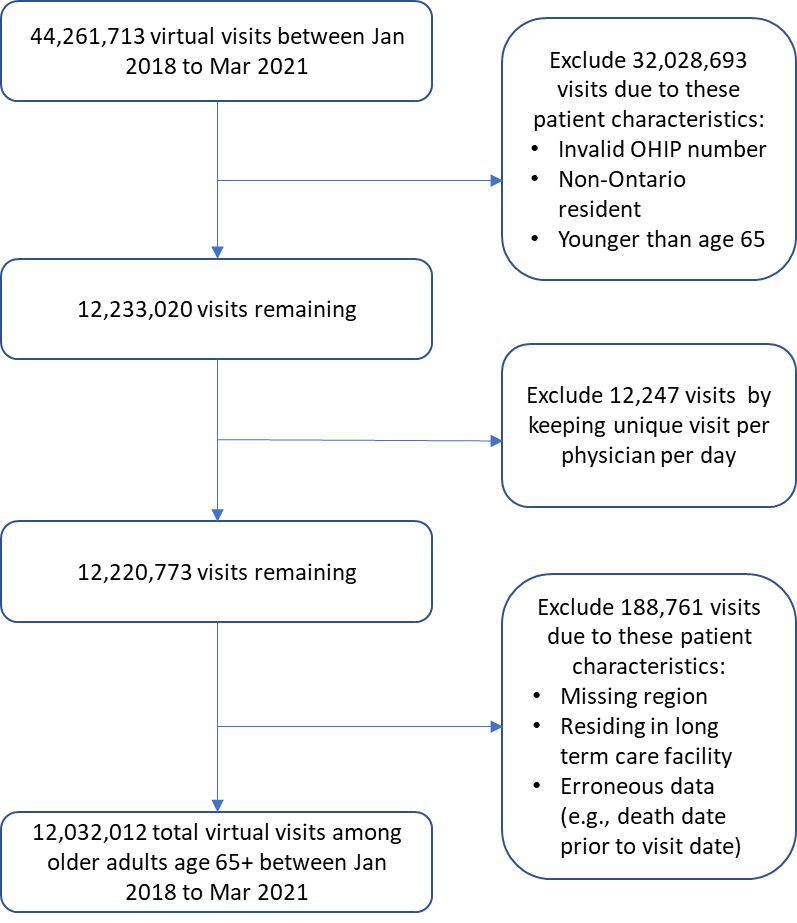
**

Supplement: S2 Appendix — (DOCX) [file pdig.0000092.s002.docx]

**S3 Appendix. Flowchart of ambulatory visits included in study**

**
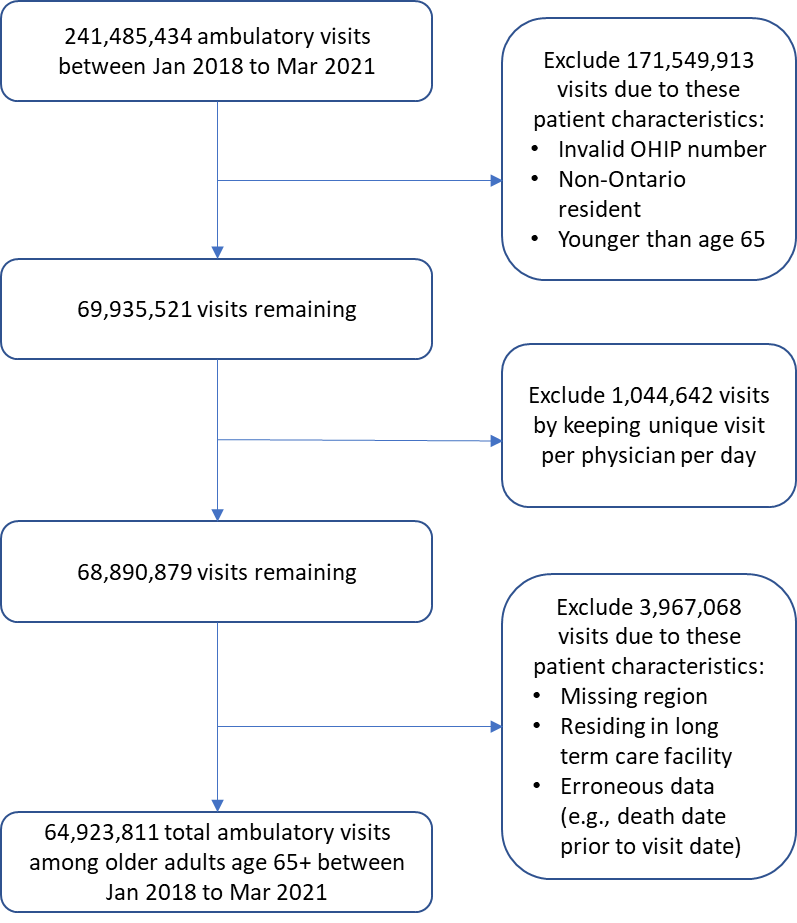
**

Supplement: S3 Appendix — (DOCX) [file pdig.0000092.s003.docx]

**S4 Appendix. Proportion of virtual vs in-person visits out of all ambulatory visits, by subgroup**

**
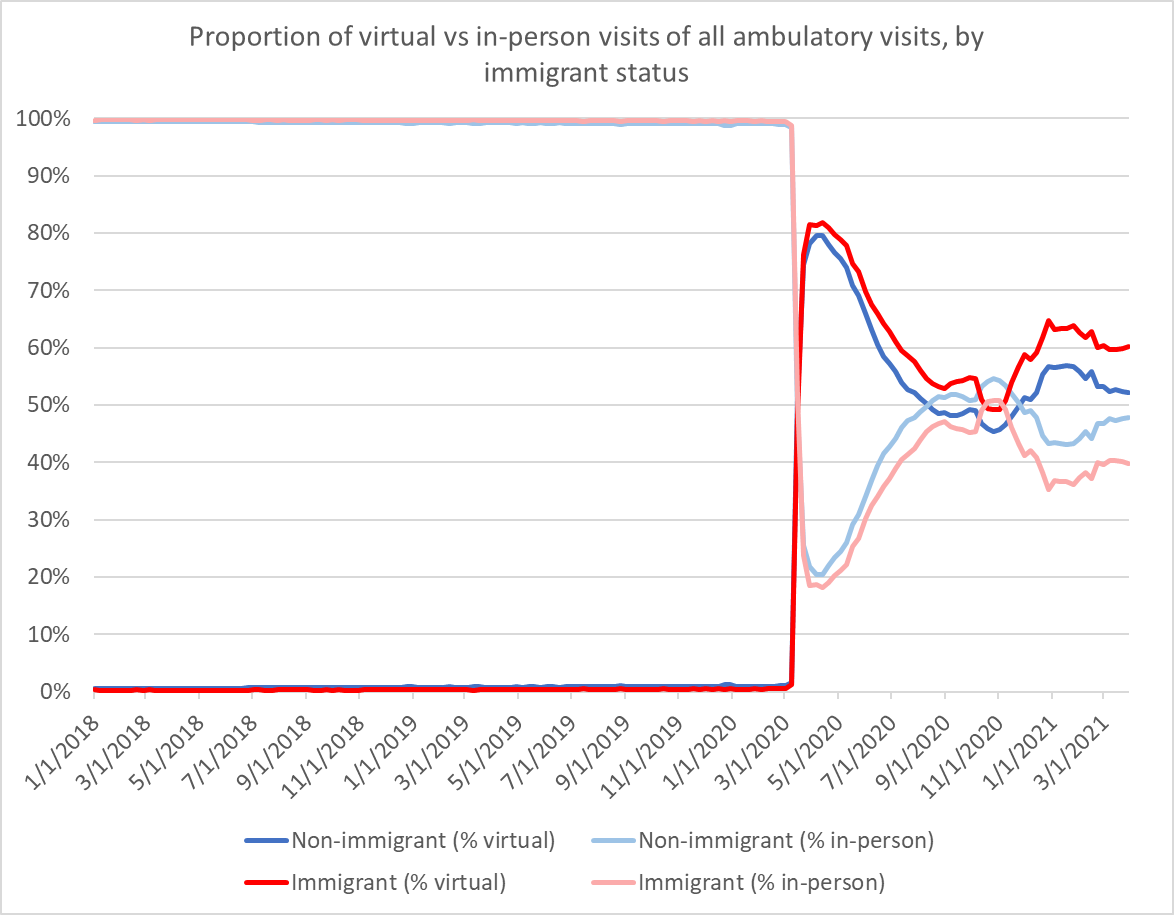
**

**
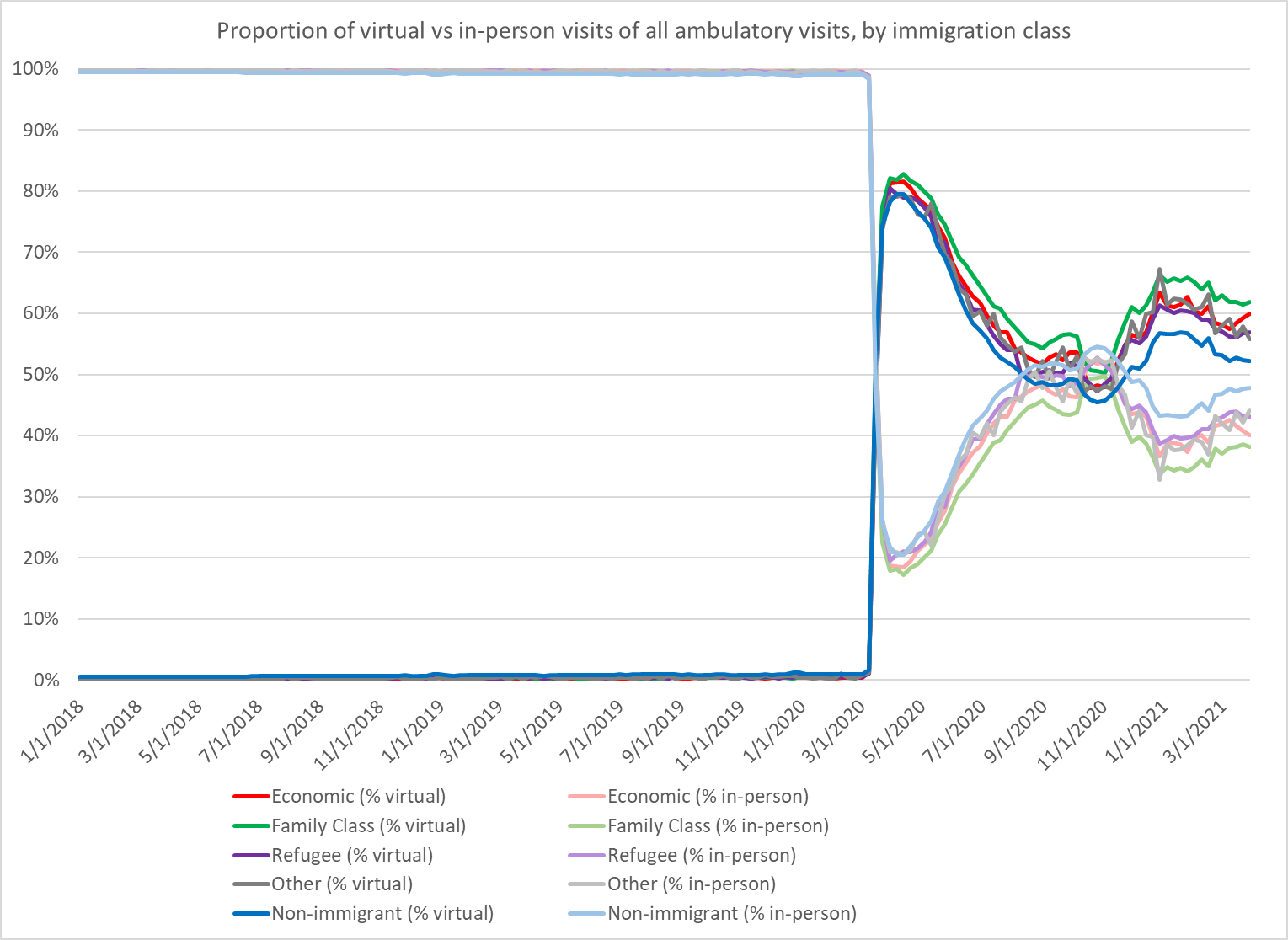
**

**
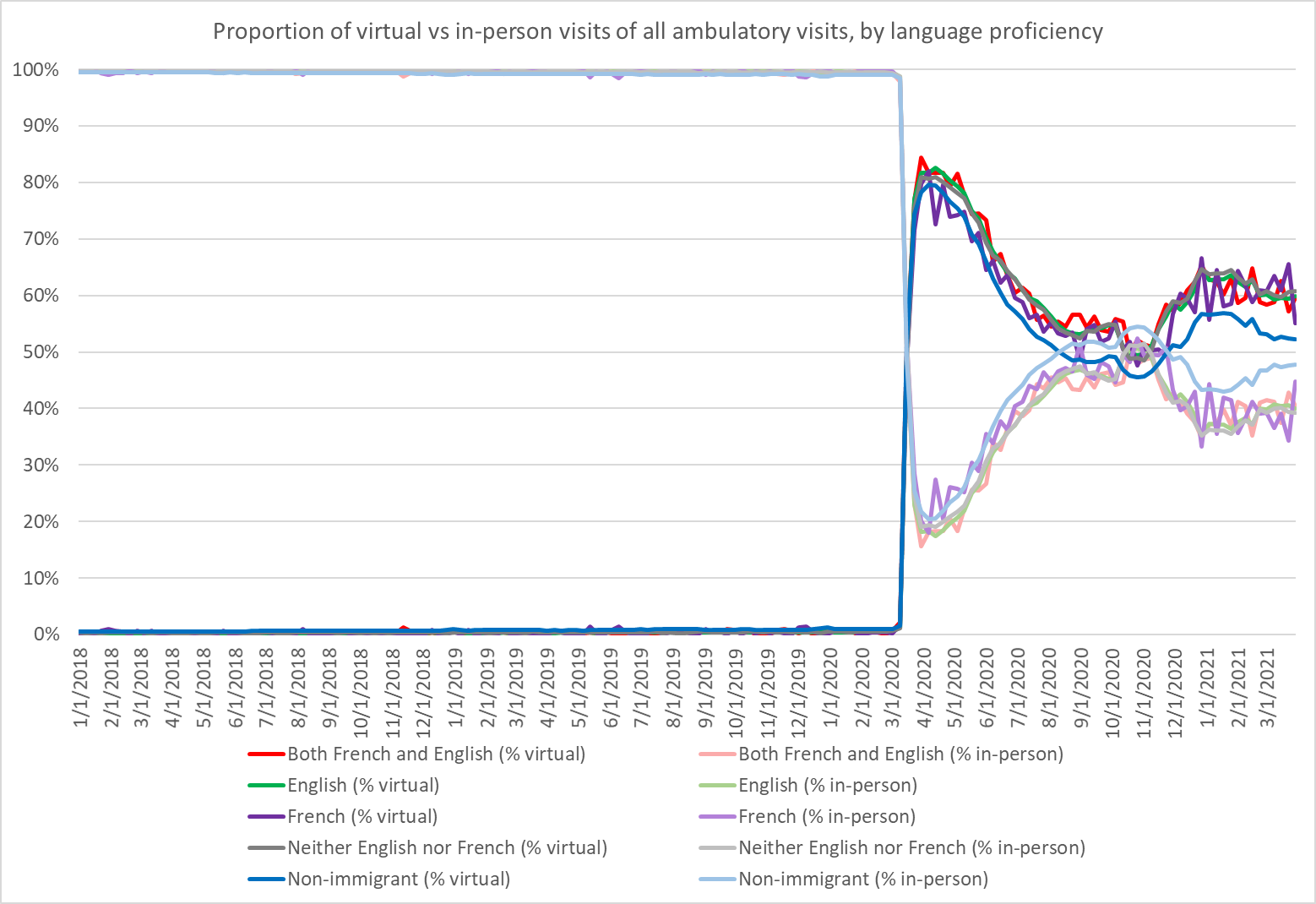
**

Supplement: S4 Appendix — (DOCX) [file pdig.0000092.s004.docx]
